# Leishmaniasis Direct Agglutination Test: Using Pictorials as Training Materials to Reduce Inter-Reader Variability and Improve Accuracy

**DOI:** 10.1371/journal.pntd.0001946

**Published:** 2012-12-13

**Authors:** Emily R. Adams, Diane Jacquet, Gerard Schoone, Kamlesh Gidwani, Marleen Boelaert, Jane Cunningham

**Affiliations:** 1 Royal Tropical Institute (Koninklijk Instituut voor de Tropen), Amsterdam, The Netherlands; 2 Institute of Tropical Medicine (ITM), Antwerp, Belgium; 3 Banaras Hindu University, Varanasi, India; 4 UNICEF/UNDP/World Bank/World Health Organization Special Programme for Research and Training in Tropical Diseases (TDR), Geneva, Switzerland; Institute of Tropical Medicine, Belgium

## Abstract

**Background:**

The Direct Agglutination Test (DAT) has a high diagnostic accuracy and remains, in some geographical areas, part of the diagnostic algorithm for Visceral Leishmaniasis (VL). However, subjective interpretation of results introduces potential for inter-reader variation. We report an assessment of inter-laboratory agreement and propose a pictorial-based approach to standardize reading of the DAT.

**Methodology:**

In preparation for a comparative evaluation of immunochromatographic diagnostics for VL, a proficiency panel of 15 well-characterized sera, DAT-antigen from a single batch and common protocol was sent to nine laboratories in Latin-America, East-Africa and Asia. Agreement (i.e., equal titre or within 1 titer) with the reading by the reference laboratory was computed. Due to significant inter-laboratory disagreement on-site refresher training was provided to all technicians performing DAT. Photos of training plates were made, and end-titres agreed upon by experienced users of DAT within the Visceral-Leishmaniasis Laboratory-Network (VL-LN).

**Results:**

Pre-training, concordance in DAT results with reference laboratories was only 50%, although agreement on negative sera was high (94%). After refresher training concordance increased to 84%; agreement on negative controls increased to 98%. Variance in readings significantly decreased after training from 3.3 titres to an average of 1.0 titre (two-sample Wilcoxon rank-sum (Mann-Whitney) test (z = −3,624 and p = 0.0003)).

**Conclusion:**

The most probable explanation for disagreement was subjective endpoint reading. Using pictorials as training materials may be a useful tool to reduce disparity in results and promote more standardized reading of DAT, without compromising diagnostic sensitivity.

## Author Summary

Until the 1990's accurate Visceral Leishmaniasis (VL) diagnosis necessitated parasitological confirmation by microscopy or culture of the blood, bone-marrow, lymph nodes or spleen. These techniques are invasive and splenic aspirates are associated with a risk of serious bleeding. This has led to the development of non-invasive serological tests such as the direct agglutination test (DAT). During infection with VL, circulating antibodies are produced against the surface antigens of the invading parasites. The DAT detects antibodies to *L. donovani s.l.* in the blood or serum of those infected by means of direct agglutination. In the absence of antibodies to *Leishmania* the DAT antigen accumulates at the bottom of the plate to form a dark blue spot. If antibodies to *Leishmania* are present then the antigen forms a pale blue film over the well constituting a positive result. Here, we report on shared experiences of six endemic countries using DAT to characterize performance panel samples. There was considerable inter-reader variability and in order to standardize the reading of DAT we developed and implemented pictorial training aids. After refresher training, agreement between readers increased; the pictorial aids and recommendations for using DAT are available in this article.

## Introduction

Up until the 1990's accurate visceral leishmaniasis (VL) diagnosis necessitated parasitological confirmation by microscopy or culture of the blood, bone-marrow, lymph nodes or spleen [1]. Microscopic detection of parasites in clinical material from the spleen is still considered the reference standard; however, splenic aspirates are associated with risk of serious bleeding and should only be carried out in settings with access to blood transfusion and surgical services. The invasiveness and potentially fatal complications associated with splenic aspiration has spurred the development of non-invasive serological tests such as direct agglutination test (DAT) [2] over 25 years ago and in the past decade, lateral flow immuno-chromatographic tests (ICT), commonly referred to as rapid diagnostic tests (RDTs). RDTs have now been adopted widely, in the Indian subcontinent [3], but in other endemic regions, DAT is part of the diagnostic algorithm or is used for epidemiological surveys due to variable sensitivity of RDTs [2], [4].

The DAT, in its present form, is a freeze dried suspension of trypsin-treated fixed and stained culture of *L. donovani* promastigotes [5]; liquid formulations of DAT are also manufactured locally. During infection with VL, circulating antibodies are produced against the surface antigens of the invading parasites. The DAT detects antibodies to *L. donovani s.l.* in the blood or serum of those infected by means of direct agglutination. In the absence of antibodies to *Leishmania* the DAT antigen accumulates at the bottom of the plate to form a dark blue spot. If antibodies to *Leishmania* are present then the antigen forms a pale blue film over the well and this constitutes a positive result.

DAT requires moderate technical expertise, and laboratory equipment and reagents, including calibrated pipettes, micro-titre plates, multiple reagents and a toxic solution (chemical 2-beta Mercapto-ethanol (2-ME)) [2]. Furthermore, despite very good accuracy, inter-observer discrepancy in routine DAT serology readings is common [6]–[8].

Prompted by shared experiences of six endemic countries using DAT to characterize performance panel samples, we report an assessment of DAT inter-reader variability. It was noted that the inter-laboratory agreement of DAT titres on a panel of 15 sera was low. Here, our objective was to standardize the reading of DAT by developing and implementing pictorial training aids.

## Materials and Methods

### Proficiency panel

Nine laboratories from three global endemic regions were involved in a WHO/TDR-sponsored evaluation of VL RDTs; namely Asia (n = 4), South America (n = 2) and Eastern Africa (n = 3) (Table 1). The Institute of Tropical Medicine, Antwerp, Belgium (ITM) assembled a proficiency panel including sera from 10 VL confirmed patients including a range of DAT titres, and 5 VL negative patients, one healthy endemic control and others who harbored potentially cross-reacting, infections, including Chagas disease, tuberculosis, malaria and leprosy. Prior to shipping, each sample within the panel was assigned a random numerical code that varied from centre to centre.

**Table 1:** Evaluation centres.

Region	Country	Institution
Participating laboratories:		
East Africa	Sudan	Faculty of Medicine, University of Khartoum
	Sudan	Institute Endemic Diseases, University of Khartoum
	Kenya	Kenya Medical Research Institute
South America	Brazil	Instituto de Medicina Tropical de São Paulo
	Brazil	Centro de Pesquisas Réné Rachou,, Fiocruz
Indian subcontinent	India	Rajendra Memorial Research Institute of Medical Sciences
	India	Institute of Medical Sciences, Banaras Hindu University
	Nepal	B P Koirala Institute of Health Sciences
	Bangladesh	International Centre for Diarrhoeal Disease Research
Reference Laboratories:	Netherlands	Royal Tropical Institute (KIT)
	Belgium	Institute Tropical Medicine (ITM)

### Ethical approval

All samples were left over from samples that had been taken as part of research projects conducted between 1978 and 2000 at the Institute for Tropical Medicine (ITM) Antwerp, Belgium. The samples were anonymised and kept stored for future use for scientific purposes. In the studies conducted since 2000 explicit consent was asked for storage and future use of left overs of the samples that were taken. In the older studies no explicit mention was made of future use of the stored left overs though a general informed consent was asked. However, it was not possible to trace back the study participants in the studies preceding 2000 and to ask them for informed consent for storage and use of left over samples

### DAT procedure

The proficiency panel was tested blindly using the DAT assay (KIT-Biomedical Research, Lot 0904) in each of the nine evaluation laboratories (Table 1) and both reference laboratories (KIT and ITM). Results were returned electronically to ITM using a standard recording form. All microtitre plates used in the procedure were provided by reference laboratories (Greiner 651101 100). The DAT was performed as described previously [2].

### Protocol development

Due to significant discordance in end-titres between all laboratories, photographs of DAT plates with 10 VL positive serum samples and 5 VL negative serum samples were prepared by the reference laboratories (following joint agreement on end titres) and were used as pictorial training aids. Refresher DAT training was given by staff of KIT and Banaras Hindu University (BHU) to all participating laboratories (Table 1). Trainers assessed equipment and compliance with the DAT SOP, including preparation of reagents. Subsequently, the proficiency panel was repeated in the presence of the trainer. End titres were read independently by two separate technicians and the trainer. When readers did not agree on the end titre they came to a common conclusion after joint discussion. Combined results of the readers were sent to ITM and decoded by a study team member not involved in the refresher training; results of the trainer were not taken into consideration unless the results of the readers were significantly different from those of the reference laboratories and the test was repeated. Disagreement was defined as greater than one titre above or below those of the reference laboratories [6]. Variance in results before and after refresher training was compared with a two-sample Wilcoxon rank-sum (Mann-Whitney) test.

## Results

Despite having received the same panels, batch of DAT, microtitre plates and protocol, overall DAT results concordance (agreement within one titre) with the reference laboratories was only 50%. Agreement on negative controls was very good (94%). Using a cut off of 1∶1600 serum dilution, the pre-training sensitivity and specificity were 79% and 94%, respectively.

Refresher training was initiated due to the large differences in DAT reading between participating laboratories. Here, photographs of DAT plates were used as training aids, where end-titres had been agreed upon by the reference laboratories. During refresher training the trainers did not identify any faulty or inappropriate equipment, nor did they witness any non-compliance with the DAT SOP. After refresher training the concordance (agreement with one DAT titre) increased to 84% with the reference laboratories. The agreement on negative controls increased to 98%. Average variance in results before refresher training was 3.3 titres; this improved to an average variance of 1.0 titre reading (the accepted limit) after refresher training. A non-parametric test was used to test for significant differences before and after training using a two-sample Wilcoxon rank-sum (Mann-Whitney) which showed significant difference (z = −3,624 and p = 0.0003). Post-training the sensitivity increased to 97% and the specificity to 100% (cut off values 1∶1600).

Overall, the refresher training increased the operator performance of the DAT in this small proficiency panel (Table 2). After refresher training a cut-off point of 1∶1,600 (serum dilution) gave 97% sensitivity (CI: 91.6–99.0%) and 100% specificity.

**Table 2:** Clinical accuracy of DAT test in proficiency panel pre and post refresher training; comparison of two cut-off titres (1∶1,600 and 1∶3,200 serum dilution).

	TP	FP	FN	TN	Sensitivity [95% CI]	Specificity [95% CI]
**Pre-training**						
Cut-off titre 1∶1,600 (Serum dilution)	80	2	21	48	0.79 [0.703 to 0.86]	0.96 [0.865 to 0.989]
Cut-off titre 1∶3,200 (Serum dilution)	74	2	27	48	0.73 [0.639 to 0.809]	0.96 [0.865 to 0.989]
**Post-training**						
Cut-off titre 1∶1,600 (Serum dilution)	98	0	3	50	0.97 [0.916 to 0.99]	1.00
Cut-off titre 1∶3,200 (Serum dilution)	95	0	6	50	0.94 [0.876 to 0.972]	1.00

Each reading at every laboratory (n = 10) is treated as a separate sample in this analysis. There are 151 total readings of the DAT as all but one centre tested the same sera; in the final centre one serum sample was replaced with another and also tested by ITM, hence 151 readings.

TP = True positive, FN = false Negative, FP = False Positive, TN = True Negative, CI = Confidence Intervals.

**Figure 1:**
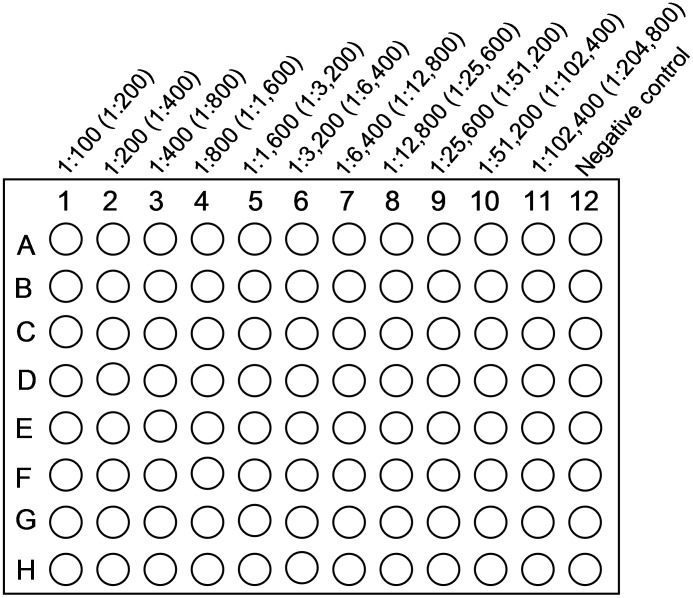
Set-up of plate. Serum dilution is given with serum (plus antigen) dilution in parentheses – it is essential to specify the type of dilution used when reporting DAT results.

**Figure 2:**
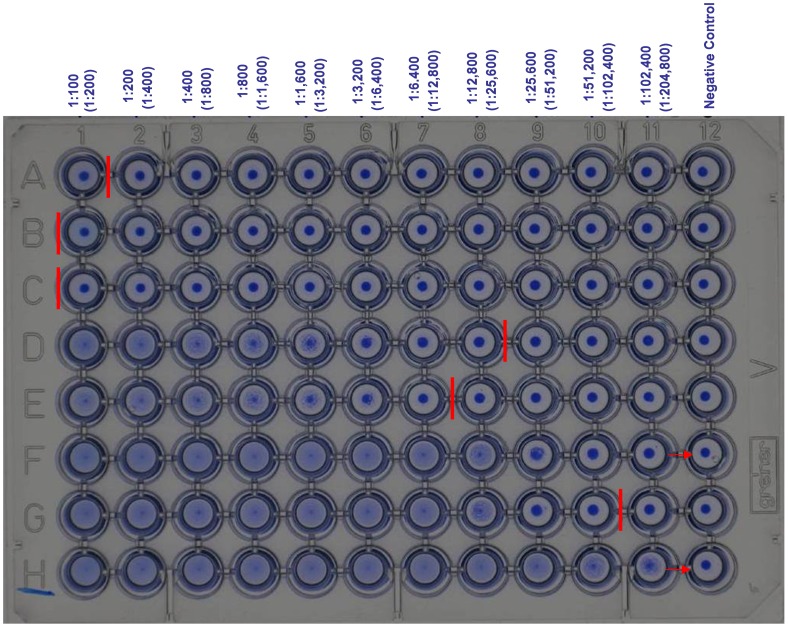
Photograph of training plate with end-titres defined by vertical red line. Where arrows show (row F &H) the end titre can not be calculated without further diluting the serum sample over two plates. Serum dilution is given with (serum plus antigen dilution) in *parentheses*.

**Figure 3:**
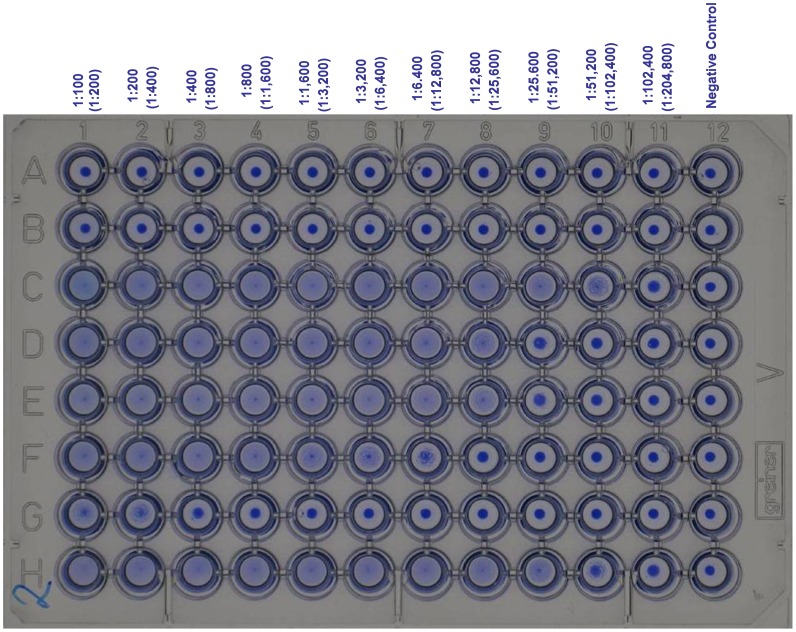
Photograph of training plate without end-titres for training purposes. Serum dilution is given with (serum plus antigen dilution) in *parentheses*. A, <1∶ 100; B, <1∶100; C, >1∶102,400; D, 1∶51,200; E, 1∶51,200; F, 1∶12,800; G, 1∶800; H, >1∶102,400 (Serum dilution).

Further, pictorial guides (Figures 1, 2, and 3) of DAT training plates reflecting consensus end titres by several experts in the VL-LN, with many years of experience in using DAT as a diagnostic tool, are now available. Further recommendations to be taken into account for completion of the DAT assay are highlighted in Table 3 and data per laboratory pre-training and post-training with pictorial aids can be seen in Supporting Information S1.

**Table 3:** Recommendations from VL-LN for DAT assay.

Consideration points	Recommendation
End Titre Reading	Reading of end titre should be last well before the one with identical dot/appearance of negative control
Pipetting	Ensure all pipettes calibrated and technician has experience with multi-channel pipettes (if used).
Controls	If available the negative control should be negative serum, preferably from endemic region
	Positive control should be included with every test
Water	Saline should be made with distilled/ultra-pure water
Plates	V-shaped plates(Greiner: 651101 100)
	Cover plates whilst test is run to avoid evaporation
Staff	Two fully trained staff should independently read the DAT plate
Antigen Batch	Try to use the same lot of antigen for each project or epidemic
Reference Sera	Try to keep reference sera to test for lot-to-lot variation for routine diagnostic work
Type of dilution	It is essential to specify the type of dilution used when reporting DAT results ie. Serum dilution or serum and antigen dilution

## Discussion

The DAT assay has been used as a diagnostic tool for more than 25 years, it is robust, reliable has a high clinical accuracy and can be performed in laboratories with minimal equipment. However, the subjective manner in which the result (end-titre) of the test is read means that inter-reader variation in titre reading can be an issue. Preparations for a multicenter evaluation of RDTs unexpectedly uncovered a significant discordance in DAT results among reference and evaluation centres; this presented an opportunity to address discordance and create an international, consensus-based protocol and training materials to strengthen standardized reading of the DAT for VL diagnosis without compromising diagnostic accuracy.

The reasons for all of the discrepancies between the different laboratories is not fully understood however, it was noted that technicians were generally competent in the DAT procedure, particularly those who used it as part of their routine diagnostic algorithm. It was not possible to test the saline that the laboratories previously used in testing, but it is possible that the origin and quality of the saline solutions used as a diluent for the DAT antigen did affect performance, generating false positive precipitation in negative sera wells. The most consistent problem identified in the laboratories can be attributed to the subjective manner in which the end titre of the DAT test is typically read. Some readers record the end-titre when 50% of agglutination of the well has occurred (as occurs with other agglutination tests), whilst other readers record the end-titre where the whole well has agglutinated and there is no difference between a negative control well (antigen plus saline) and the sample well. Even though 1 titre difference in reading is considered acceptable [6], the discrepancy and variance in results reported here was far greater. Training plates developed by the reference laboratories proved to be extremely helpful in illustrating the end titre. Positive sample wells were defined by any reaction in the test well in comparison to the negative control well; this ensured that the high sensitivity of the DAT was not compromised. Figure 2 shows the end-titre as agreed by the VL-LN; a follow up plate can be used to test users before revealing the results as seen in figure 3. High quality photos in figures 2 and 3 are also available by request (contact corresponding author) for use as reference training material for future DAT users.

Since slight variations in readings between different DAT antigen batches may occur it is advised that the same batch of DAT should be used within one project or epidemic to decrease variability in results. If this is not possible then it is recommended to keep reference sera in order to assess this lot-to-lot variation, this should not be more than one titre difference. In addition, it is important that all users of the DAT specify the type of dilution used, i.e. serum dilution (starting 1∶100) or antigen plus serum dilution (starting 1∶200). It is likely that cut-off values are different between endemic areas and even during epidemic cycles. Local guidance as to appropriate cut-off values is essential.

The problems uncovered during a multicenter DAT proficiency testing scheme are potentially relevant to other DAT users. To reduce inter-reader variability and increase accuracy, photos of training plates were made, and end-titres were agreed upon firstly by the reference laboratories and subsequently by experienced users of DAT within the VL-LN. These photos can be used to promote a standardized approach to interpreting DAT without compromising sensitivity. Protocols and photos can be requested for training and quality control purposes by two of the major manufacturers of the assay, KIT and ITM. High sensitivity and specificity can be achieved with this reliable and robust diagnostic tool, and we hope that provision of good training materials can increase the usefulness of DAT.

## Supporting Information

Supporting Information S1 Table 4a: Results per laboratory before refresher training. The number expressed is the well number of the last well where a positive reaction is seen. Table 4b: Results per laboratory post-training with pictorial materials. The number expressed is the well number of the last well where a positive reaction is seen.
(DOCX)
