# Supplementary material for: Leishmaniasis Direct Agglutination Test: Using Pictorials as Training Materials to Reduce Inter-Reader Variability and Improve Accuracy
Source: PLoS Negl Trop Dis. 2012 Dec 13;6(12):e1946. doi: 10.1371/journal.pntd.0001946 (PMC3521667; doi:10.1371/journal.pntd.0001946)
Supplement: Supporting Information S1 — Table 4a: Results per laboratory before refresher training. The number expressed is the well number of the last well where a positive reaction is seen. Table 4b: Results per laboratory post-training with pictorial materials. The number expressed is the well number of the last well where a positive reaction is seen. (DOCX) [file pntd.0001946.s001.docx]

Supporting Information S1

Table 4a: Results per laboratory before refresher training. The number expressed is the last well where a positive reaction is seen.

Table 4b: Results per laboratory post-training with pictorial materials. The number expressed is the last well where a positive reaction is seen.
